# Modulation of redox homeostasis under suboptimal conditions by Arabidopsis nudix hydrolase 7

**DOI:** 10.1186/1471-2229-10-173

**Published:** 2010-08-12

**Authors:** Niranjani Jambunathan, Anuradha Penaganti, Yuhong Tang, Ramamurthy Mahalingam

**Affiliations:** 1 246 Noble Research Center, Department of Biochemistry and Molecular Biology, Oklahoma State University, Stillwater, Oklahoma, USA; 2 The Samuel Roberts Noble Foundation Inc., Plant Biology Division, Ardmore, Oklahoma, USA

## Abstract

**Background:**

Nudix hydrolases play a key role in maintaining cellular homeostasis by hydrolyzing various nuceloside diphosphate derivatives and capped mRNAs. Several independent studies have demonstrated that *Arabidopsis nudix hydrolase 7 *(AtNUDT7) hydrolyzes NADH and ADP-ribose. Loss of function *Atnudt7-1 *mutant plants (SALK_046441) exhibit stunted growth, higher levels of reactive oxygen species, enhanced resistance to pathogens. However, using the same T-DNA line, two other groups reported that mutant plants do not exhibit any visible phenotypes. In this study we analyze plausible factors that account for differences in the observed phenotypes in *Atnudt7*. Secondly, we evaluate the biochemical and molecular consequences of increased NADH levels due to loss of function of AtNUDT7 in Arabidopsis.

**Results:**

We identified a novel conditional phenotype of *Atnudt7-1 *knockout plants that was contingent upon nutrient composition of potting mix. In nutrient-rich Metro-Mix, there were no phenotypic differences between mutant and wild-type (WT) plants. In the nutrient-poor mix (12 parts vermiculite: 3 parts Redi-earth and 1 part sand), mutant plants showed the characteristic stunted phenotype. Compared with WT plants, levels of glutathione, NAD^+^, NADH, and in turn NADH:NAD^+ ^ratio were higher in *Atnudt7-1 *plants growing in 12:3:1 potting mix. Infiltrating NADH and ADP-ribose into WT leaves was sufficient to induce AtNUDT7 protein. Constitutive over-expression of *AtNudt7 *did not alter NADH levels or resistance to pathogens. Transcriptome analysis identified nearly 700 genes differentially expressed in the *Atnudt7-1 *mutant compared to WT plants grown in 12:3:1 potting mix. In the *Atnudt7-*1 mutant, genes associated with defense response, proteolytic activities, and systemic acquired resistance were upregulated, while gene ontologies for transcription and phytohormone signaling were downregulated.

**Conclusions:**

Based on these observations, we conclude that the differences observed in growth phenotypes of the *Atnudt7-1 *knockout mutants can be due to differences in the nutrient composition of potting mix. Our data suggests AtNUDT7 plays an important role in maintaining redox homeostasis, particularly for maintaining NADH:NAD^+ ^balance for normal growth and development. During stress conditions, rapid induction of AtNUDT7 is important for regulating the activation of stress/defense signaling and cell death pathways.

## Background

Pyridine nucleotides (PNs), which include NAD^+^, NADP^+^, NADH and NADPH, are ubiquitous coenzymes involved in redox reactions in all organisms [1,2]. In plants, PNs act as developmental cues during the process of seed germination [3] and for transitioning from the vegetative to reproductive state [4]. Levels of PNs in plants are altered by light conditions and age of plants [5]. Changes in PN level in response to abiotic stresses like chilling and drought or in response to fungal elicitors and pathogens have also been reported [6-9]. Recently, it has been shown that extracellular PNs induce pathogenesis-related (PR) gene expression and disease resistance pathways in Arabidopsis [10]. Thus, PN homeostasis impacts several developmental and stress signaling pathways in plants.

Several studies in animal systems have shown that the ratio of oxidized to reduced form of PNs, especially NAD^+^: NADH, acts as an important signal that connects metabolic states of the cell to its gene expression pattern [11-13]. Cellular PN levels, or more importantly, redox states, are sensed by repressors of gene expression, which in turn regulate chromatin architecture [11,14]. However, the enzymes regulating PN levels and in turn its impact on gene expression have not been well studied in plants.

Nudix (nucleoside diphosphates linked to moiety X) hydrolases play a vital role in cellular homeostasis by catalyzing the hydrolysis of a variety of nucleoside diphosphate derivatives including NADH, NAD^+^, ADP-ribose, NTPs, dNTPs, phosphoinositol derivatives, and capped mRNAs [15]. Since these substrates have regulatory roles or may be toxic, nudix hydrolases play a key role in signaling and house-cleaning processes. There are 29 nudix hydrolases identified in *Arabidopsis thaliana *[16]. In vitro enzymatic analyses have been carried out for nine cytosolic nudix hydrolases of Arabidopsis [17]. The first characterized plant nudix hydrolase, *AtNUDT1*, was NADH pyrophosphatase [18]. *AtNUDT1 *was later shown to be the canonical mutT-type nudix hydrolase in Arabidopsis, important for scavenging oxidized nucleotides, especially deoxyguanosines [19]. Recently, it was shown that over-expression of AtNUDT2, an ADP-ribose pyrophosphatase, confers enhanced tolerance to oxidative stress [20]. This enhanced tolerance was attributed to maintenance of NAD and ATP levels by nucleotide recycling from free ADP-ribose under stress conditions [20]. Several independent research groups have analyzed various aspects of Arabidopsis nudix hydrolase 7 (AtNudt7) [6,17,20-25]. In vitro analysis demonstrated that AtNUDT7 could use both NADH and ADP-ribose as substrates [6,17,23,24]. Over-expression of AtNudt7 (*P_35s_: AtNUDT7*) led to a decrease in both NADH and ADP-ribose levels, whereas in a T-DNA knockout line, *Atnudt7-1*, (SALK_046441), the levels of these two metabolites were higher than wild-type (WT) plants grown under the same conditions, suggesting that both NADH and ADP-ribose are physiological substrates of this protein [22].

The knockout Arabidopsis mutant, *Atnudt7-1*, which was previously referred to as *growth factor gene 1 (gfg1*), has been described as having pleiotropic phenotypes such as reduced size, higher levels of reactive oxygen species (ROS), microscopic cell death, constitutive expression of pathogenesis-related (PR) genes, and improved resistance to a virulent bacterial pathogen, *Pseudomonas syringae *DC3000 [23]. Both *Atnudt7-1 *and an independent T-DNA knockout line, *Atnudt7-*2 (SALK_104293), were reported to exhibit reduced size, higher levels of salicylic acid, and increased resistance to an oomycete pathogen, *Hyaloperonospora parasitica *[26]. Another independent group demonstrated constitutive PR gene expression, increased resistance to a virulent bacterial pathogen, and reduced hypersensitive response to avirulent bacterial pathogens (*P syringae Avrrpt2 *and *P glycinea Avrrpt2*) in *Atnudt7-1 *mutant [21]. However, two other groups using the *Atnudt7-1 *line reported no differences in the growth or morphology of mutant plants under normal growing conditions [6].

This contradiction in reported *Atnudt7-1 *phenotype combined with similar observations in our laboratory prompted us to examine this mutant more carefully. In this study, we describe a conditional growth phenotype of *Atnudt7-1 *that is influenced by edaphic factors. Mutant plants were reduced in size when grown in nutrient-poor mix of vermiculite: Redi-earth: sand (12:3:1), but grew to the same size as WT plants when raised on nutrient-rich Metro-Mix (MM). Higher levels of AtNUDT7 protein were observed when WT plants were grown in 12:3:1 mix and under several abiotic and biotic stress conditions culminating in cell death. Interestingly, increased NADH was observed in *Atnudt7-1 *only under suboptimal growing conditions. Affymetrix gene chip analysis of *Atnudt7-1 *mutants grown under suboptimal conditions showed substantial changes in gene expression. Genes associated with systemic acquired resistance (SAR) and cell death pathways were induced in the mutant. Down regulation of several hormone-signaling pathways in the mutant indicated interconnections between PN homeostasis and phytohormones. These results demonstrate that AtNUDT7 plays a crucial role in regulating NAD^+^: NADH balance under suboptimal conditions, which in turn modulates the activation of defense, phytohormones and cell death signaling pathways.

## Results

### Atnudt7-1 mutant exhibits a conditional phenotype

**Figure 1:**
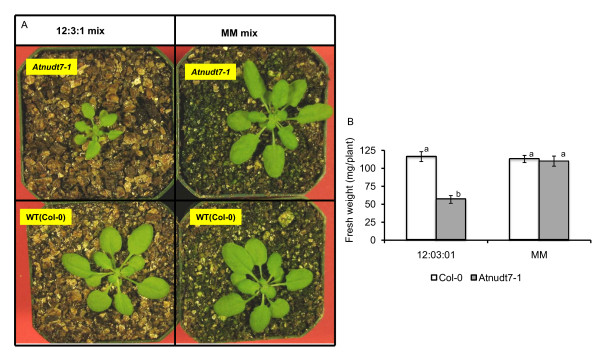
**Conditional phenotype of *Atnudt7-1 *mutant plants in different potting mixes**. A. Three-week-old WT Col-0 and *Atnudt7-1 *mutant plants grown in two different potting mixes under identical conditions. The 12:3:1 potting mix is a mix of 12 parts of vermiculite, 3 parts of Redi-earth and 1 part of sand. Metro-Mix 200 (MM) is the commercial potting mix. B. Average fresh weight of 4-week old plants grown in 12:3:1 mix and metro-mix potting soil (n = 48 plants for each condition).

We observed that the type of potting mix influenced the phenotype of *Atnudt7-1 *mutant. The typical stunted growth phenotype of *Atnudt7-1 *was observed consistently when plants were raised in 12:3:1 potting mix. *Atnudt7-1 *mutant plants in Metro-Mix 200 (MM) were the same size as WT plants (Fig. 1A and Fig. 1B). In the 12:3:1 potting mix, growth of WT Col-0 was comparable to the plants in MM for 3-4 weeks (Fig. 1A and Fig. 1B). We analyzed the nutrient composition of both mixes (Table 1). The 12:3:1 potting mix had low levels of all tested macro- and micronutrients. In some cases, the levels of these nutrients were lower than the optimum range recommended. These data suggested that the phenotype of *Atnudt7-1 *was strongly influenced by the microenvironment in which these plants were growing.

**Table 1:** Nutrient analysis of the potting mixes used for growing *Atnudt7-1 *plants

	12:3:1 mix	Metro-Mix 200
Nitrate-N (ppm)	14	68
Potassium (ppm)	25	241
Phosphorus (ppm)	3.5	42.6
Calcium (ppm)	93	290
Magnesium (ppm)	19	324
Ammonium-N (ppm)	0.4	0.6
Iron (ppm)	11.8	24.4
Zinc (ppm)	1.4	3.2
Copper (ppm)	2.7	4.1
Sulfate (ppm)	300	1043
Boron (ppm)	0.1	0.3

Supplementing individual nutrients (N, P, K, Ca, and Mg) in 12:3:1 potting mix to the levels observed in MM improved the growth habit of the mutant plants. However, compared with WT plants the mutant plants were still smaller in size (data not shown). Irrigating the mutant plants in 12:3:1 potting mix with a commercial fertilizer solution restored the WT phenotype with respect to size or biomass.

**Figure 2:**
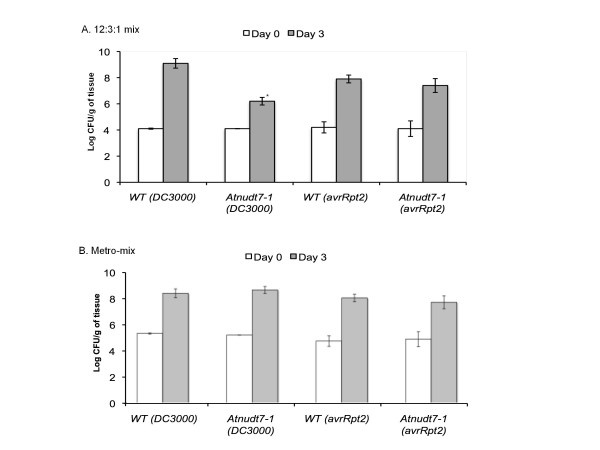
**In planta growth of *Pseudomonas syringae *in WT Col-0 and *Atnudt7-1 *plants raised on Metro-Mix**. Growth of virulent *P syringae *DC3000 and avirulent *P syringae *DC3000 containing Avrrpt2 were monitored on the day of infiltration and three days after infiltration. Error bars represent average of 3 replicates ± SD. CFU stands for colony forming units.

*Atnudt7-1 *grown in 12:3:1 mix showed reduced growth of virulent bacterial pathogen *P. syringae *pathovar tomato (DC3000), indicating enhanced resistance of these plants to pathogens [6,21,23,26] (Fig. 2). When *Atnudt7-1 *plants were grown in MM the increased resistance to *P. syringae *was compromised (Fig. 2).

### AtNudt7 is upregulated in plants grown under suboptimal conditions

**Figure 3:**
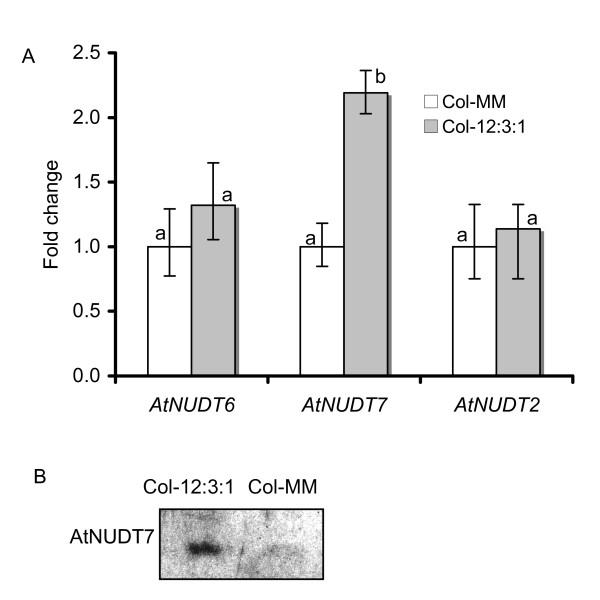
**Expression of *AtNudt7 *and its closest homologs in WT Col-0 plants growing in different potting mixes**. A. Gene expression levels of *AtNudt2, AtNudt6 *and *AtNudt7 *in WT Col-0 plants grown in 12:3:1 and Metro-Mix 200 (MM) potting mix, as determined by real-time PCR. Fold change in 12:3:1 mix was plotted with respect to expression in MM. Error bars indicate standard deviation of three replicates. Bars with different letters indicate statistically significant differences (p-value < 0.05) in gene expression in WT plants grown in 12:3:1 mix when compared with those grown on MM. B. Detection of AtNUDT7 protein in WT Col-0 plants grown in the 12:3:1 potting mix and Metro-Mix 200 (MM) using AtNUDT7 polyclonal antibodies.

AtNUDT7 protein shares close sequence homology to AtNUDT2 and AtNUDT6 [26]. *In vitro *assays indicate AtNUDT7 has affinity for ADP-ribose and NADH, the preferred substrates for AtNUDT2 and AtNUDT6 [17]. We monitored the transcriptional profiles levels of these three nudix hydrolase genes under different growth conditions. Steady-state transcript levels of *AtNudt2 *and *AtNudt6 *did not show any difference in Col-0 plants grown in the two different potting mixes. Compared to plants in MM a 2-fold up regulation of *AtNudt7 *transcripts was observed in plants grown in 12:3:1 mix (Fig. 3A).

Induction of AtNUDT7 protein was also observed in WT Col-0 plants grown in 12:3:1 mix (Fig. 3B). Accumulation of *AtNudt7 *transcripts and protein under nutrient deficient growing conditions in WT plants suggests that this protein may play an important role under these situations.

### AtNUDT7 protein levels are induced in response to biotic and abiotic stresses

**Figure 4:**
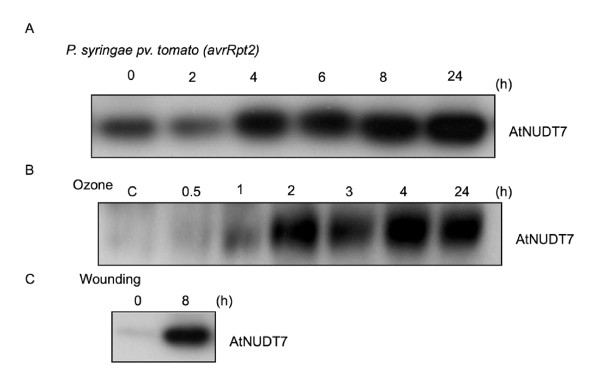
**Western analysis using polyclonal antibodies raised against recombinant AtNUDT7 protein**. A. WT Col-0 plants were infected with 1 × 10^6 ^CFU/mL of *Pseudomonas syringae avrRpt2*. Leaves were harvested immediately after infiltration (0), and at 2, 4, 6, 8, and 24 hours post-infection For each lane, 20 μg of total protein was used. B. Three-week old Wassilewskija ecotype plants were fumigated with 250 nL. L^-1 ^of ozone for six hours. Rosettes were collected at 0.5, 1, 2, 3, and 4 hours during ozone treatment and 24 hours after the initiation of treatment. Control (C) plants were maintained under ambient ozone conditions. For each lane, 20 μg of total protein was used. C. AtNUDT7 protein levels were monitored in WT Col-0 plants eight hours after wounding by a sharp blade. For each lane, 20 μg of total protein was used.

We monitored AtNUDT7 protein accumulation in Col-0 WT plants after infiltrating leaves with *Pseudomonas syringae *pathovar tomato *(avrRpt2) *bacterial pathogen, which induces the hypersensitive cell death response. AtNUDT7 protein levels had accumulated to high levels by four hours of pathogen treatment (Fig. 4A). Similarly, rapid accumulation of AtNUDT7 was observed after two hours of acute ozone treatment in the ozone sensitive Ws-0 ecotype (Fig. 4B). We also observed that AtNUDT7 protein levels were induced within eight hours of wounding by a sharp blade (Fig. 4C). These data clearly demonstrated rapid induction of AtNUDT7 protein during both biotic and abiotic stress treatments that culminate in cell death.

### Over-expressing AtNUDT7 does not alter PN levels but alters expression of closely related nudix hydrolases

**Figure 5:**
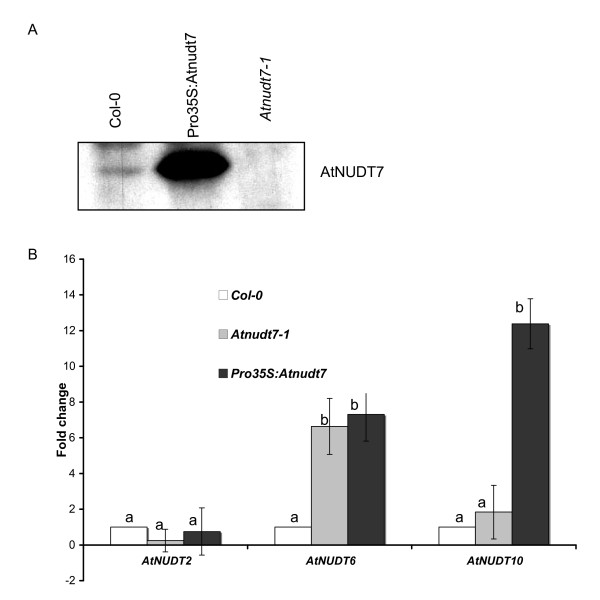
**Analysis of *P_35S_:AtNudt7 *transgenic lines in Col-0 background**. A. AtNUDT7 protein levels in Col-0, *Pro_35S_:AtNudt7 *transgenic lines and *Atnudt7-1 *mutant plants based on western blot analysis with anti-NUDT7 antiserum. B. Expression of *AtNUDT2, AtNUDT6 *and *AtNUDT10 *genes in Col-0, *Pro_35S_:AtNUDT7 *and *Atnudt7-1 *mutant plants by real-time PCR analyses. Error bars indicate standard deviation of three replicates. Bars with different letters indicate statistically significant differences in gene expression when compared with the corresponding WT.

Transgenic plants (T2 generation) that over-express AtNUDT7 under the control of constitutive cauliflower mosaic 35 S promoter (*P_35S_: AtNudt7*) were generated in the WT Col-0 background and confirmed by western analysis using AtNUDT7 polyclonal antibodies (Fig. 5A). The *P_35S_:AtNudt7 *plants were similar to WT in size in both MM and 12:3:1 mix. *P_35S_:AtNudt7 *plants infiltrated with *P. syringae *DC3000 did not show any differences in pathogen growth compared with WT plants (Additional file 1, Fig. S1). Levels of NAD and NADH did not change in *Pro_35S_:AtNudt7 *plants when compared with the vector control or with WT Col-0 plants grown in MM or 12:3:1 mix (Additional file 1, Fig. S1). Another group over-expressing the *AtNudt7 *gene under the control of 35 S promoter reported similar results [6].

Using real-time PCR, we monitored the expression levels of the closest homologs of *AtNudt7 *in over-expressor lines and compared them to those of the *Atnudt7-1 *mutant and WT plants (Fig. 5B). Expression of *AtNudt6 *and *AtNudt10 *genes was higher in *P_35S_: AtNudt7 *plants than WT. Interestingly, *AtNudt6 *was also highly expressed in *Atnudt7-1 *mutant plants. The expression of *AtNudt2 *was not altered in the *P_35S_:AtNudt7 *and *Atnudt7-1 *plants.

### Induction of AtNUDT7 protein is triggered by its substrates

**Figure 6:**
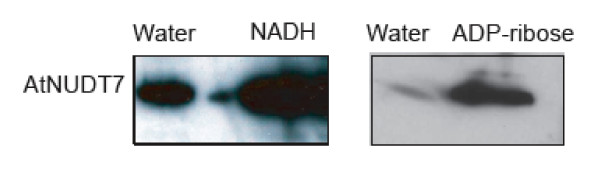
**Infiltration of NADH or ADP-ribose into leaves induces AtNUDT7 protein**. Approximately 5 mM NADH or 1 mM ADP-ribose in water was infiltrated into leaves of WT Col-0 plants. Control plants were infiltrated with water. Treated leaves were harvested 24 hours after treatment and used for western blot analysis.

Exogenous application of NADH or ADP-ribose to WT plants led to a substantial increase in AtNUDT7 protein levels when compared to control plants infiltrated with water (Fig. 6). Based on these observations, we speculate that NADH and ADP-ribose are likely physiological substrates for AtNUDT7 or these metabolites may confer stability to this protein.

### Redox perturbations under suboptimal conditions are plausible triggers for inducing AtNUDT7 protein

**Figure 7:**
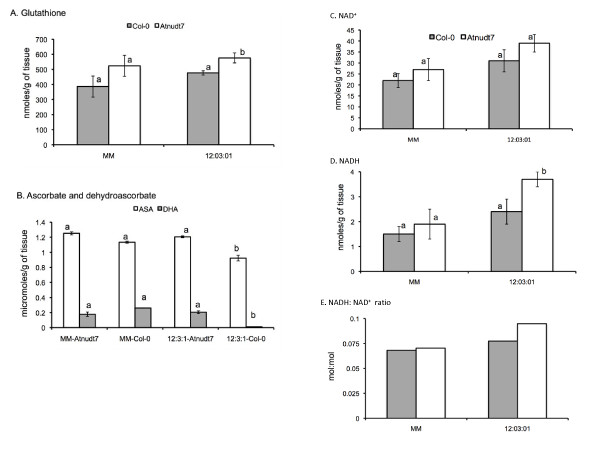
**Cellular redox balance is affected in *Atnudt7-1 *mutant**. WT Col-0 and *Atnudt7-1 *mutant plants grown in 12:3:1 and Metro-Mix 200 (MM) potting mix were used for these analyses. A. Glutathione. B. Reduced ascorbate and dehydoascorbate. C. NAD^+^. D. NADH. E. Ratio of NADH: NAD^+^. Error bars represent standard deviation derived from two biological experiments with three replicates in each experiment. Bars with different letters indicate statistically significant differences in the measured metabolite in the *Atnudt7-1 *mutant compared with the corresponding WT.

Glutathione levels were higher in *Atnudt7-1 *mutant plants than WT plants grown under similar conditions, which was again consistent with an earlier study [6]. Differences in glutathione levels in WT plants grown in the two different potting mixes were not significant (Fig. 7A).

Amount of ascorbate (AsA) and dehydroascorbate (DHA) were similar in WT and *Atnudt7-1 *plants grown in MM. AsA levels in *Atnudt7-1 *mutant growing in 12:3:1 potting mix were comparable to those observed in MM. Interestingly, we observed nearly 30% decrease in the AsA levels of WT plants grown in 12:3:1 mix (Fig. 7B). Furthermore, DHA levels were almost undetectable in the WT plants growing in 12:3:1 mix. This indicated that differences in potting mix caused significant changes in AsA/DHA redox couple in WT plants, even though their consequences did not manifest phenotypically.

Comparison of NAD^+ ^levels in WT and *Atnudt7-1 *plants growing in MM or in 12:3:1 mix did not show any significant differences (Fig. 7C). NAD+ levels in plants growing in 12:3:1 mix was higher than in plants growing in MM but was not statistically significant for *Atnudt7-1*. *Atnudt7-1 *plants in 12:3:1 mix showed almost 2-fold higher levels of NADH than WT plants (Fig. 7D). In contrast, *Atnudt7-1 *plants in MM did not show any change in NADH levels compared to WT plants grown under identical conditions. The observed increase in NAD^+ ^and NADH levels in *Atnudt7-1 *plants growing in 12:3:1 mix manifested as higher NADH: NAD^+ ^ratios when compared to plants growing in MM (Fig. 7E). These data showed that alterations in growth conditions including nutrient status impact NADH: NAD^+ ^ratios in plants, and lack of AtNUDT7 protein exaggerated the changes in this redox couple.

### Substantial changes in gene expression in *Atnudt7-1 *plants grown under suboptimal conditions

Based on studies in animal systems, we speculated that higher levels of NADH and GSH in *Atnudt7-1 *plants under suboptimal conditions might bring about changes in gene expression. Arabidopsis ATH1 gene chips with 22,500 probe sets representing 24,000 genes were used to examine changes in transcript levels in *Atnudt7-1 *plants with respect to expression observed in WT controls grown in 12:3:1 mix. Experiments were conducted with 3-week-old plants since the phenotype of the mutant plants was distinct at this stage of development when grown in 12:3:1 potting mix. Based on two biological replications of the Genechip experiments (R^2 ^= 0.98), 1607 genes were reliably detected in the WT versus *Atnudt7-1 *comparison. There were 396 genes that were 2-fold induced and 470 genes that were 2-fold repressed (Additional file 2, Table S1). Thus, under suboptimal growing conditions, lack of AtNUDT7 protein resulted in extensive changes in gene expression.

**Figure 8:**
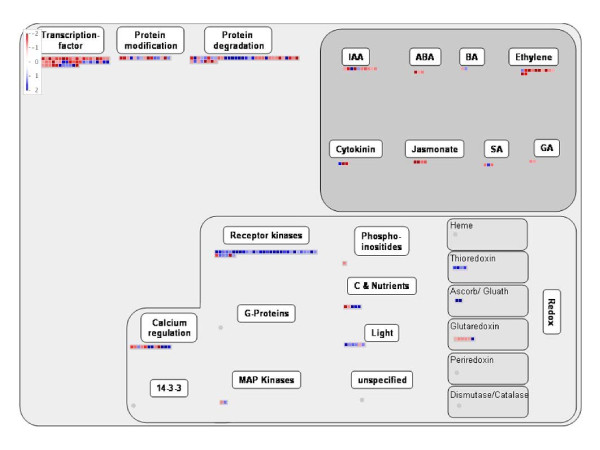
**Genes differentially expressed in the *Atnudt7-1 *mutant under the gene ontology of regulation**. Each box represents a gene; blue box indicates induction and red indicates repression of gene expression in *Atnudt7-1 *plants compared to WT Col-0 grown in 12:3:1 potting mix. This figure was generated using the MAPMAN software. IAA, auxins; BA, brassinosteroids; ABA, abscisic acid; SA, salicylic acid; GA, gibberellic acid; Ascorb/Gluath, ascorbate/glutathione.

To gain insight into the biological significance of the genes differentially expressed in *Atnudt7-1*, we used MAPMAN analysis [27]. The overrepresented gene ontologies are presented in Table 2. Marked changes in the expression of genes regulating transcription, protein degradation, signaling, redox and phytohormones were seen in the *Atnudt7-1 *mutant (Fig. 8). The majority of the transcription factor encoding genes (37) was repressed, while those induced belonged to the WRKY family. The enrichment of GO for transcription among the repressed genes is supportive of the role of PNs as global metabolic regulators of gene expression [28]. Identification of a large set of genes induced in response to biotic stress provides a molecular basis for the increased resistance to pathogens reported for *Atnudt7-1 *[6,23]. Four different thioredoxin genes were upregulated while four of the five differentially expressed glutaredoxins were down regulated (Fig. 8), suggesting that the redox network is perturbed, perhaps due to imbalances in the redox input elements. Down regulation of ethylene, auxin, jasmonate responsive genes and ABA biosynthesis genes were observed in *Atnudt7-1 *mutant. A gene encoding isochorismate synthase, important for salicylic acid (SA) biosynthesis [29], was up regulated, which may account for reported increase in SA levels in the *Atnudt7-1 *mutant [26].

**Table 2:** Overrepresented gene ontologies in *Atnudt7-1 *mutant in comparison with WT plants grown in 12:3:1 potting mix as determined by MAPMAN software

GO category	p-value
Secondary metabolism-flavonoids	0.002
Signaling-receptor kinases	0.002
Biotic stress	0.005
Regulation of transcription	0.015
Unknown	0.033
Hormone metabolism-ethylene	0.038
Protein degradation	0.043

**Figure 9:**
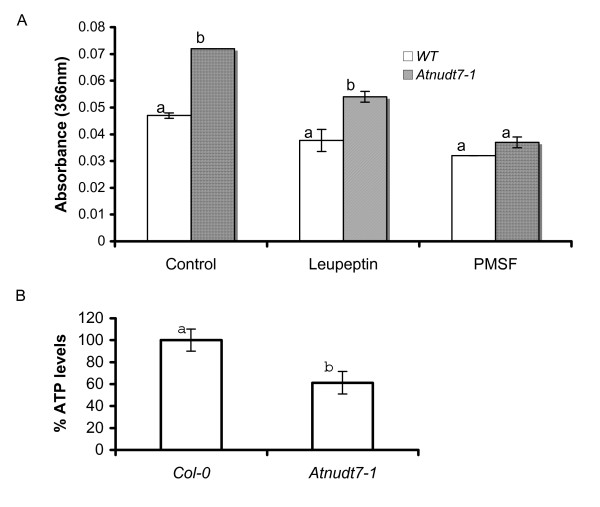
**Biochemical assays to support the microarray based gene expression data in the *Atnudt7-1 *mutant**. A. Protease activity assay in WT Col-0 and *Atnudt7-1 *mutant plants using azocasein as substrate. Leupeptin (0.1 mM) and phenylmethyl sulfonate (PMSF; 1 mM) were added to protein extracts from WT and mutant plants. The assay was repeated four times, twice from each of the two biological replicates of plants. Bars with different letters indicate statistically significant differences in protease activity compared with WT. B. Analysis of ATP content in WT Col-0 and *Atnudt7-1 *plants. The assay was repeated four times, twice from each of the two biological replicates of plants. Bars with different letters indicate statistically significant differences in ATP levels in *Atnudt7-1 *mutant compared with WT.

The observed enrichment of genes involved in proteolysis led us to examine the total protease activity in the mutant plants. Protein extracts from *Atnudt7-1 *leaves exhibited significantly higher protease activity using azocasein as substrate, providing biochemical corroboration for the microarray data (Fig. 9A). Addition of phenylmethanesulfonyl chloride, an inhibitor of Ser proteases and papain family cysteine proteases [30], or leupeptin, an inhibitor of cysteine proteases, reduced protease activity in both *Atnudt7-1 *and WT plants. There was a significant reduction in protease activity in *Atnudt7-1 *plant extracts after the addition of PMSF, indicating that serine proteases contributed most to the proteolytic activity.

**Figure 10:**
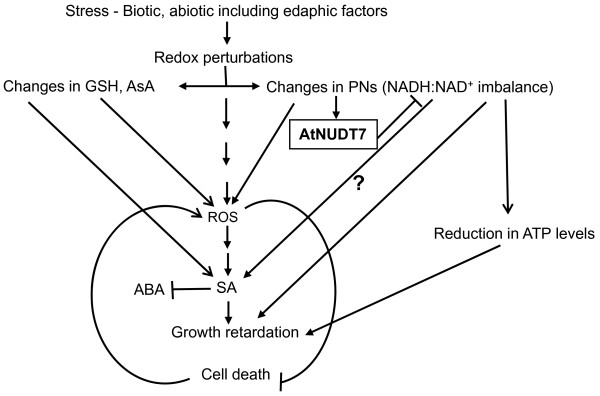
**Working model for AtNUDT7 protein, linking redox and oxidative cell death cycles**. Biotic and abiotic stresses including suboptimal growing conditions lead to alterations in ascorbate, GSH and PNs. Changes in NADH induces AtNUDT7 and maybe crucial to restore the balance of NAD^+^:NADH redox couple. In the absence of AtNUDT7 protein, NADH levels increase and trigger the accumulation of SA by an unknown mechanism and activate the SAR pathway, which in turn can antagonize ABA signaling. SA accumulation can trigger cell death via ROS, which in turn can inhibit the cell death promotive SA signal. Reduction in ATP levels in *Atnudt7-1 *may be due to constitutive activation of defense/stress signaling that in turn may lead to growth retardation.

The induction of signaling genes, especially receptor-like kinases (RLKs) in the *Atnudt7-1 *mutant, is intriguing (Additional file 3, Fig. S2). This observation suggested that changes in NAD: NADH balance maybe sufficient to activate a number of RLKs important for transducing signals to downstream mediators. The significant reduction in the ATP levels of *Atnudt7-1 *mutant suggests energy metabolism is compromised when PN balance is perturbed (Fig. 9B). The reported increases in SA levels and the gamut of phytohormone responsive genes and redox transmitters that are altered in the *Atnudt7-1 *mutant in conjunction with the observed changes in NAD^+^, NADH, GSH and ascorbate, suggests novel interconnections between redox signaling, antioxidative systems and phytohormone-mediated oxidative cell death pathways [31] (Fig. 10).

## Discussion

In this study, we report a conditional phenotypic response to an edaphic factor in *Atnudt7-1 *mutant plants (Fig. 1). In the two earlier reports indicating that *Atnudt7-1 *did not show any obvious phenotypes, mutant plants were grown in soil that was fertilized [6] or were grown on nutrient rich MS plates [22]. Loss of stunted growth phenotype in *Atnudt7-1 *plants grown in nutrient rich metro-mix is consistent with these earlier reports. Restoration of WT phenotype in the *Atnudt7-1 *plants grown in 12:3:1 mix supplemented with fertilizers demonstrated that this mutant is hyper-responsive to the growth micro-environment.

Poor nutrient levels of 12:3:1 mix (Table 1) were sufficient to induce AtNUDT7 protein in WT plants, indicating that expression of this protein is important under such suboptimal conditions (Fig. 3). This is supported by strong and rapid accumulation of AtNUDT7 protein under conditions of pathogen, wounding and ozone stress (Fig. 4). Although the transcript levels of *AtNudt7 *showed only transient induction for a few hours following stress treatments [23], the protein was stable for 24 hours (Fig. 4A-C). The half-life prediction of 30 hours for AtNUDT7 by the ProtParam program of ExPASy supports this observation. It is also possible that some posttranslational modification could confer stability to the AtNUDT7 protein.

Lack of AtNUDT7 protein resulted in elevated resistance to bacterial and oomycete pathogens [6,21,23,26]. However, our studies showed that the resistance to pathogens was compromised when *Atnudt7-1 *mutants were raised in MM potting soil (Fig. 2). Over expression of AtNUDT7 protein in WT plants did not alter their phenotype nor alter growth of virulent and avirulent *P. syringae *pathogens (Additional file 1, Fig. S1). Based on these observations, we argue that the increased resistance to pathogens observed in *Atnudt7-1 *plants is an indirect effect of the mutation.

Over-expression of AtNUDT7 led to elevated transcript levels of closely related nudix hydrolases (Fig. 5) suggesting coordinate regulation of members of this gene family or regulation via the substrates on which these enzymes act. Microarray data comparing *Atnudt7-1 *and WT plants indicated transcript levels of several nudix hydrolases were altered. This included *AtNudt5 *and *AtNudt6 *that were induced, while expression of *AtNudt8*, *AtNudt17 *and *AtNudt24 *were repressed (Additional file 2, Table S1). In the light of these observations, we caution that analysis of pyrophosphohydrolase activities in transgenic lines overexpressing or silencing particular nudix hydrolases may be misleading.

In vitro studies of AtNUDT7 have revealed that this protein has significant pyrophosphohydrolase activity with NADH and ADP-ribose [17,23,24], and has recently been confirmed using transgenic lines [22]. Inducibility of AtNUDT7 protein by NADH and ADP-ribose provides indirect evidence that these metabolites may be its physiological substrates (Fig. 6). However, the primary biochemical function of nudix hydrolase may not be the hydrolysis of its substrates [6]. It may be involved only in conformational change as demonstrated for LTRP2, a nudix box containing protein, important for mediating calcium influx to trigger immune responses in animal immunocytes [32,33].

*Atnudt7-1 *mutant in 12:3:1 potting mix had 2-fold higher levels of NADH than WT plants grown under the same conditions (Fig. 7). In the male-sterile CMSI mutant of *Nicotiana sylvestris*, deficient in the mitochondrial complex I, levels of NAD^+ ^and NADH were 2-fold higher than WT plants [34] and interestingly these plants showed growth retardation similar to *Atnudt7 *mutant. Interestingly, increase in NADH levels did not cause changes in ROS levels in CMSII mutant but lead to reorchestration of antioxidative mechanisms resulting in higher tolerance to ozone and viral infections [35]. In another Arabidopsis mutant deficient in cytosolic glycerol-3-phosphate dehydrogenase (*gpdhc1*), increases in NADH levels and in turn the NADH: NAD^+ ^ratio led to enhanced ROS levels under standard growing conditions and significantly augmented H_2_O_2 _production under stress [36,37]. Higher levels of NADH were reported in *Atnudt7-1 *mutants six hours after infection with *P. syringae *(Avrrpt2) pathogen [6]. In protein analysis experiments following *P. syringae *(Avrrpt2) pathogen infection, we observed strong induction of AtNUDT7 protein at a comparable time point (Fig. 4). This suggests under pathogen-imposed stress conditions NADH levels increase and AtNUDT7 may be required to restore NAD^+^: NADH balance. Such changes in NAD^+ ^and NADH levels during stress can lead to redox imbalance, triggering the production of ROS via one-electron reduction of oxygen [8,36,38] or may be important for orchestrating cellular antioxidant systems [35]. The higher ROS levels observed in *Atnudt7-1 *[23,26] may be causally connected to the increase in NADH and/or NADH: NAD^+^.

In the *Atnudt7-1 *mutants exhibiting reduced size phenotype, levels of SA were reported to be 4- to 5-fold higher than WT [26]. Several other SA-overproducing mutants have also been reported to exhibit a growth retardation phenotype [39]. The role of SA in SAR against pathogens is well known [40-42]. Furthermore, SA and ROS induce each other and form a feed-forward self-amplifying loop [43-45]. The observed increase in ROS levels in the *Atnudt7-1 *mutant could be due to increased levels of SA.

This begs the question - why are SA levels high in the *Atnudt7-1 *mutant? We speculate that an increase in SA levels may be brought about in response to redox perturbations. A positive correlation between an increase in SA levels and a corresponding increase in GSH levels using constitutive SA accumulating mutants, as well as by exogenous application of these metabolites, has been reported [46]. Increasing the levels of GSH by transgenic approaches or chemical intervention was sufficient to mimic induction of SA response genes [47-49]. The earlier reported increases in SA [26] and observed increases in GSH (Fig. 7) in *Atnudt7-1 *are consistent with the aforementioned studies. Recently, it was reported that exogenous application of NAD(P) induced pathogenesis-related genes and resistance to *P. syringae maculicola*, as well as accumulation of SA [10]. It is tempting to speculate that increased NADH levels or redox perturbations caused by changes in NADH:NAD^+ ^favoring the reductant, trigger the production and/or accumulation of physiologically coupled SA, GSH and ROS in *Atnudt7-1 *plants, that in turn leads to the observed pleiotropic phenotypes.

One of the other pleiotropic phenotype associated with *Atnudt7-1 *is microscopic cell death [23,26]. The observed constitutive activation of proteolysis-associated genes in microarray analysis (Additional file 2, Table S1) and confirmation of enhanced proteolytic activity in *Atnudt7-1 *plant extracts (Fig. 9A) provides a plausible explanation for the cell death phenotype. We speculate that the cell death phenotype is a manifestation of the interplay between the phytohormones, ROS and proteolysis related genes [50].

PNs are key redox input elements in the regulatory thiol-disulfide network [51] and have important roles in pro-oxidant and antioxidant metabolism [52]. PNs have also been observed to play major roles in non-redox mechanisms that influence cell function. NAD(H) is considered a key modulator of cellular energy metabolism [53,54]. Significant reduction in ATP levels of *Atnudt7-1 *mutant supports these earlier observations (Fig. 9B). Several lines of evidence in animal systems suggest that NAD(H) also mediates cell death [55-57], calcium homeostasis [1,54] and gene expression [14,58]. Substantial transcriptome alterations (especially the down regulation of GO category transcription) observed in *Atnudt7-1 *plants are in agreement with reports in the animal literature (Fig. 8). Changes in PNs and GSH can also play an important role in gene regulation via components such as Non-Expressor of PR1 (NPR1) protein [59,60].

Antagonistic cross-talk occurs at multiple levels between the SA-mediated signaling of the SAR pathway and the ABA-mediated responses to abiotic stress [61]. Microarray analysis revealed that several abiotic stress signaling pathways were down regulated, in the *Atnudt7-1 *mutant. This is in agreement with reports of antagonistic interactions between the SA-mediated defense in response to pathogens and ABA signaling pathways [62,63].

## Conclusions

Constitutive expression of AtNUDT7 under normal growing conditions is important for maintaining NAD^+^: NADH homeostasis. Stress, in the form of nutrient deficiency, pathogens, wounding or ozone, causes rapid and transient alterations in AtNUDT7 protein levels that can in turn alter the redox balance. These redox perturbations are important for coordinating changes in signaling pathways, including those of phytohormones, ROS, antioxidants and cell death. The mechanisms leading to an increase in SA levels due to changes in PN levels are currently unknown. It has been suggested that sporadic cell death can lead to leakage of PNs to the extracellular space, which can trigger SA biosynthesis and downstream defense signaling [10]. This increase in SA then leads to a buildup of ROS in a feed-forward self-amplifying loop, leading to an activated SAR pathway. Activation of SAR in turn leads to down regulation of abiotic stress signaling pathway mediated by ABA. Induced ROS can serve as a cue to suppress SA promotive signal in the propagation of cell death [64]. However, the sporadic cell death in the *Atnudt7-1 *mutant did not lead to confluent lesions supporting the notion that AtNUDT7 normally restricts initiation rather than propagation of cell death [26]. Based on our studies, it is clear that AtNUDT7 is an important player linking redox metabolism and oxidative stress signaling (Fig. 10). Understanding the regulation of *AtNUDT7 *expression will facilitate the identification of transcription factors crucial for redox homeostasis in plants both during normal growth and under stress conditions.

## Methods

### Plant materials

Seeds of WT, transgenic (*P_35S_: AtNudt7*), and mutant (*Atnudt7-1*) lines were stratified in 0.1% Phytagel (Caisson Laboratories) at 4°C for 3 days. Seeds were sown in pots containing either a mixture of 12 parts vermiculite, 3 parts Redi-earth (composition: 55-65% sphagnum peat moss, vermiculite, dolomitic lime; Sun Gro Horticulture) and 1 part sand (12:3:1) or Metro-Mix 200 (MM) potting mix (Scotts) for 4 weeks in a growth chamber. Plants were maintained at 22°C, 45% relative humidity, 100 μmol m^-2 ^s^-1 ^light intensity and 10 h day and 14 h dark conditions. Plants were irrigated using tap water. About 12-15 entire rosettes were harvested, flash-frozen in liquid nitrogen and stored at - 80°C prior to RNA extraction. Two independently grown and harvested sets of samples were used for RNA extraction and hybridization.

### Promoter_35S_:AtNudt7 overexpressor plants

To construct transgenic plants over expressing AtNudt7, the full-length coding sequence of AtNudt7 cDNA was amplified by RT-PCR. For cloning purposes, *Xba*I and *Bam*HI sites were introduced at the 5' end of the forward primer (5'-CTAG**TCTAGA**ATGGGTACTAGAGCTCAGCA-3') and the 3' end of the reverse primer (5'-CGC**GGATCC**TCAGAGAGAAGCAGAGGCTTG-3') (restriction sites highlighted in bold), respectively. The amplified fragment was cloned into pGEM T vector and sequenced from both directions using universal primers to ensure that sequences were free of mutations. The insert was isolated by restriction digestion and cloned downstream of 35 S promoter in *Xba*I-*Bam*HI digested binary vector pSR3000. Arabidopsis Col-0 plants were transformed by *Agrobacterium tumefaciens *using the floral dipping method [65]. Transgenic plants were selected on MS medium containing 50 μg mL^-1 ^kanamycin. Protein extracts from T2 generation transgenic plants were analyzed by western analysis using AtNUDT7 polyclonal antibodies.

### Potting mix analysis

Macronutrients and micronutrients measurements in the 12:3:1 potting mix and the commercial Metro-Mix 200 were conducted at the Soil, Water and Forage Analytical Laboratory, Oklahoma State University. Micronutrients were extracted with diethylenetriaminepentaacetic acid solution (pH 7.3). Nitrates and ammonium were analyzed on a flow-injection analyzer (Lachat); the nitrates were analyzed using cadmium reduction [66] and the ammonium was analyzed using the salicylate method [67]. Ca, Mg, K, B, SO4, Fe, Zn, and Cu were analyzed directly by an inductively coupled plasma emission spectrometer (Model Ciros, Spectro).

### Stress and chemical treatments

WT Col-0 plants were grown in MM potting soil and were fertilized on a regular basis. Plants were subjected to biotic and abiotic stresses to examine the changes in AtNUDT7 protein levels.

Bacterial pathogen infection: A suspension (1x10^5 ^cfu/mL in 10 mM MgCl_2_) of virulent *Pseudomonas syringae *pv. tomato, DC3000 strain, and avirulent strain *P. syringae*, *AvrRpt2 *were infiltrated into 4-week-old WT Col-0 plants using a needleless syringe. Leaves were harvested 2, 4, 6, 8 and 24 hours after infiltration. Mock-infiltrated leaf samples (10 mM MgCl_2_) were harvested at the same time as controls. Growth curve analysis of the virulent bacteria was conducted for the plants growing in MM and 12:3:1 mix as described earlier [23].

Acute ozone treatment: WT Ws-0 ecotype plants were grown under short day conditions for 4 weeks and then exposed to 250 nL L^-1 ^of ozone for duration of six hours as described earlier [68]. Symptoms on the leaves were evaluated 24 hours after the end of ozone treatment. Leaf samples were harvested 0.5, 1, 2, 3, 4 and 24 hours after the start of treatment.

Wounding: WT Col-0 plants were grown for five weeks in MM under the same conditions described above. Fully expanded leaves were wounded using a sharp blade. About eight hours after treatment, the wounded leaves were harvested for western analysis.

### NADH and ADP-ribose treatment

Five-week-old Col-0 plants were infiltrated using a needleless syringe with 5 mM NADH (Sigma) or 1 mM ADP-ribose (Sigma) dissolved in water. Control plants were infiltrated with distilled water. Leaves from control and NADH or ADP-ribose infiltrated plants were harvested 24 hours after treatment and flash-frozen in liquid nitrogen.

### AtNUDT7 polyclonal antibodies

Polyclonal anti-NUDT7 antisera were generated in rabbits using full-length recombinant AtNUDT7 as the antigen (Pacific Immunologies Corp.). The generated antisera detected a 33 kDa protein band in total soluble protein extracts from WT Col-0 leaf tissues. This was consistent with the predicted size of AtNUDT7 protein in the Arabidopsis database.

### Western analysis

Total protein was extracted from rosettes using protein extraction buffer (50 mM Tris pH 7.5, 150 mM sodium chloride, 50 mM sucrose, 1 mM phenylmethylsulfonyl fluoride, 0.1% Triton X-100, 1 μl plant protease inhibitor cocktail). Protein concentrations were determined using Bradford reagent (Bio-Rad). Each protein sample (50 μg) was resolved on a 15% SDS-PAGE gel and transferred to a polyvinylidene difluoride membrane by electro blotting (Bio-Rad). The membrane was stained with Coomassie blue (0.2% Coomassie blue, 50% methanol, 10% acetic acid) to check for equal loading and blocked overnight at 4°C with Tris-buffered saline, pH 7.6, containing 0.1% Tween 20 and 5% skim milk. Western blot hybridization was conducted as described in the Amersham Biosciences manual. The membrane was incubated for one hour on a shaker at room temperature with a 1:2000 dilution of primary polyclonal anti-rabbit AtNudt7 antibodies (Pacific Immunology Corp.) in Tris-buffered saline, pH 7.6, containing 0.1% Tween 20 and 5% skim milk. Following washes, membranes were incubated for one hour in secondary ECL anti-rabbit IgG horseradish peroxidase-linked whole antibody (Amersham Biosciences) at a dilution of 1:25,000 in TBST. Signal was detected using an ECL kit (Amersham Biosciences).

### RNA isolation, GeneChip hybridization, and data analysis

Total RNA was isolated using the Plant RNeasy kit (Qiagen) from two independent biological replicates of *Atnudt7-1 *and WT plants growing in 12:3:1 mix. RNA was precipitated using sodium acetate and ethanol. RNA quality and quantity was assessed in a Bioanalyzer (Agilent Technologies). Approximately 10 μg of total RNA was used for hybridization, as described earlier [69].

For each Affymetrix array hybridized, the resulting .cel file was exported from GeneChip Operating Software Version 1.4 (Affymetrix) and imported into Robust Multiarray Average [70] for global normalization. The presence/absence call for each probe set was made using dCHIP software [71]. Gene selections based on an associative t-test [72] were made using Matlab software (MathWorks, Natick, MA). Using this method, the background noise present between replicates and technical noise during hybridization were measured by the residual presented among a group of genes. Only genes whose residuals between the compared sample pairs were significantly higher than the measured noise level were considered differentially expressed. Since the residual was obtained from thousands of genes on the chip, the p-value obtained by this method was corrected for a large sampling size, thus allowing the use of Bonferroni corrections without being overly stringent. The advantage of this methodology is that it takes into consideration technical noise and internal variation between replicates within a sample group and provides a baseline for selecting biologically significant genes [73]. A selection threshold of 2 for transcript ratios (where applicable) and a Bonferroni-corrected p-value threshold of 2.19202E-06 were used to select genes for pathway reconstruction using MAPMAN software [27]. The Bonferroni-corrected P = 0.05/N, where N is the number of genes in the comparison (22,810 in the experiments reported here). The microarray data have been submitted under the accession number E-MEXP-2711 to the ArrayExpress database.

### Protease activity assay

Azocasein was used as substrate in a general protease assay [68] using total protein extracts from *Atnudt7-1 *and WT plants. Phenylmethylsulfonyl fluoride (1 mM), an inhibitor of the Ser proteases and the papain family Cys proteases, and leupeptin (0.1 mM), a well-known inhibitor of Cys proteases, were added separately to the homogenate. The assays were replicated three times.

### ATP measurement

About 100 mg of leaf tissue was homogenized with 10 mM Tris-HCl buffer (pH 7.2). Following centrifugation at 20,000*g *for 10 min at 4°C, the supernatant was collected for ATP analysis. ATP was determined using an ATP Bioluminescent Assay kit (Sigma FL-AA). The assays were performed in 15 mm × 60 mm vials with a luminometer (Lumac/3 M Biocounter M2010A) preset to integrate the amount of light produced over a 10 second interval without an initial delay. Levels of light produced in WT Col-0 and *Atnudt7-1 *plant extracts were used to estimate the amount of ATP based on standards. ATP content of the control plants was set to 100% to determine the relative ATP content.

### NAD^+^/NADH measurements

PN measurements were conducted using 0.2 g of ground leaf tissue using the enzyme cycling assay [36,74]. The analysis was repeated four times, twice from each biological replicate. Standard curves for NAD^+ ^and NADH were generated each time the assay was conducted.

### Ascorbate and glutathione measurements

Ascorbate and glutathione in WT and *Atnudt7-1 *mutant plants grown in 12:3:1 soil mix and MM were measured as described earlier [68]. To measure ascorbate, about 0.2 g of leaf tissue was first ground to a fine powder and resuspended in 2 mL of 2% metaphosphoric acid and 2 mM EDTA. Following neutralization with 10% sodium citrate, total ascorbate was analyzed by measuring change in absorbance on a spectrophotometer (PerkinElmer) before and after addition of ascorbate oxidase [75]. To measure glutathione, the neutralized plant extract was mixed with 6.3 mM 5',5'-dithiobis-2-nitrobenzoic acid, 5 mM NADPH and one unit of glutathione reductase and then incubated for six minutes at room temperature. Absorbance at 412 nm was recorded [76]. Two independent measurements each from two biological replications were averaged and used for plotting the graphs.

### Real-time PCR analysis

Total RNA was diluted to 200 ng/μL. Approximately 1 μg of this RNA was used for cDNA synthesis by SuperScript reverse transcriptase (Invitrogen) according to the manufacturer's instructions. Primer sequences for all the genes used in RT-PCR analysis are provided in the supporting information (Additional file 4, Table S2). Primers were designed using the Primer Express program (Applied Biosystems) to amplify an 80 base pair fragment. Real-time RT-PCR was performed using the SYBR Green kit (Fermentas) as described earlier [77]. Samples were run and analyzed using an ABI PRISM 5700 (Applied Biosystems) according to the manufacturer's instructions. The experiment was repeated twice using different cDNA preparations and the average delta delta Ct values were plotted with standard deviation.

### Statistical analysis

One-way ANOVA was performed to study the differences in NAD^+^, NADH, GSH, AsA and DHA levels in *Atnudt7-1 *mutant and WT Col-0 plants grown in two different potting mixes (MM and 12:3:1 mix). Tukey's studentized range test (95% confidence level) was performed using Statistical Analysis Software (SAS Enterprise Group 4.0, SAS Institute). Microsoft Excel was used for calculating statistical significance of real-time PCR data between two treatments or samples.

## Authors' contributions

NJ conducted all the biochemical assays, phenotype analysis, real-time PCR and drafted the manuscript. AP conducted the microarray experiments and western analysis. YT conducted microarray data analysis. RM was responsible for overseeing the experiments, data verification and writing the manuscript. All authors have read and approved the final manuscript.

## Supplementary Material

Additional file 1 **Fig. S1: Analysis of bacterial growth and pyridine nucleotide levels in P_35S_: AtNUDT7 transgenic plants**. Click here for file

Additional file 2 **Table S1: List of genes that are differentially expressed in *Atnudt7-1 *mutant compared with WT plants grown in the 12:3:1 potting mix**. Click here for file

Additional file 3 **Fig. S2: MAPMAN view of receptor-like kinases altered in the *Atnudt7-1 *mutant**. Click here for file

Additional file 4 **Table S2: List of primers used for real-time PCR analysis**. Click here for file
