# Supplementary material for: Modulation of redox homeostasis under suboptimal conditions by Arabidopsis nudix hydrolase 7
Source: BMC Plant Biol. 2010 Aug 12;10:173. doi: 10.1186/1471-2229-10-173 (PMC3095304; doi:10.1186/1471-2229-10-173)
Supplement: Additional file 1 — Fig. S1: Analysis of bacterial growth and pyridine nucleotide levels in P35S: AtNUDT7 transgenic plants. [file 1471-2229-10-173-S1.PDF]

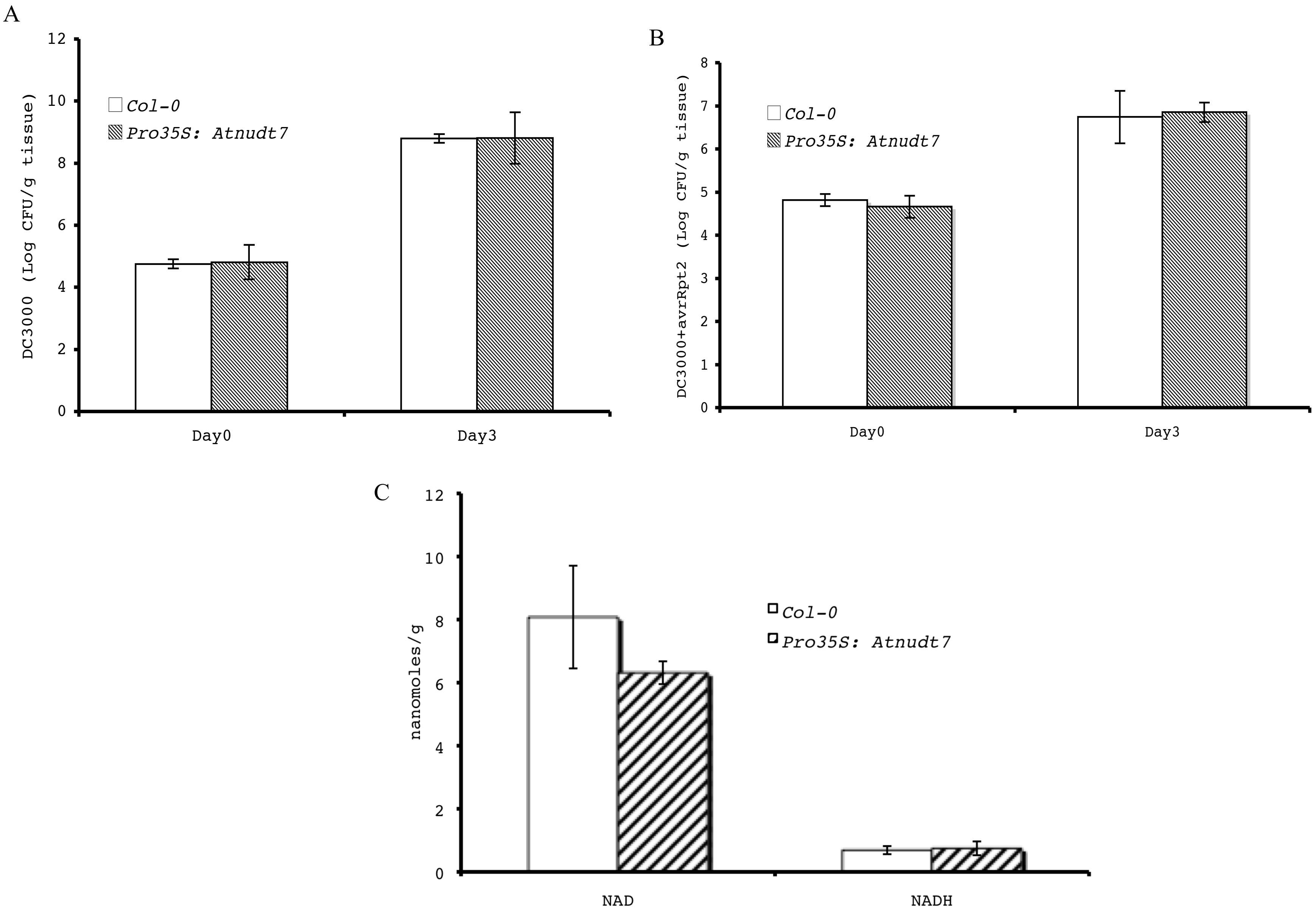

Fig S1: Analysis of Pro35S:AtNUDT7 in Col-0 background. A. Growth of virulent bacteria (P Syringae DC3000) in over-expressor and WT plants. B. Growth of avirulent bacteria (P syringae DC 3000+Avrrpt2) in over-expressor and WT plants. C. NAD and NADH analysis in over-expressor and WT plants. For each experiment the average of 3 replicates is shown. Error bars represent SD.
