# Supplementary material for: Modulation of redox homeostasis under suboptimal conditions by Arabidopsis nudix hydrolase 7
Source: BMC Plant Biol. 2010 Aug 12;10:173. doi: 10.1186/1471-2229-10-173 (PMC3095304; doi:10.1186/1471-2229-10-173)
Supplement: Additional file 2 — Table S1: List of genes that are differentially expressed in Atnudt7-1 mutant compared with WT plants grown in the 12:3:1 potting mix. [file 1471-2229-10-173-S2.PDF]

Table S1. Description of differentially expressed genes in AtNudt7-1 versus Wild-type plants growing in 12:3:1 potting mix

Target Description	Public ID	Atnudt7/WT
putative Fe(II)/ascorbate oxidase SRG1 protein	At4g10500	36.32
similarity to DNA-binding protein	At5g22570	23.55
beta-1,3-glucanase 2 (BG2) (PR-2)	At3g57260	19.81
serine/threonine kinase -KIK1 of maize	At4g23150	18.39
putative protein CGI-131 protein	At4g21830	19.11
putative tyrosine aminotransferase	At2g24850	15.60
AIG1	At1g33960	21.69
hypothetical protein predicted by genscan	At2g18660	18.24
S-receptor kinase PK3 precursor	At5g18470	3.81
putative WRKY-type DNA binding protein	At2g46400	2.57
calcium-binding protein - like cbp1	At5g39670	4.14
putative disease resistance protein Hcr9-9A	At4g13900	4.86
similar to unknown protein	At5g52760	5.59
disease resistance protein RPP1-WsB	At1g57630	5.18
putative receptor-like protein kinase	At4g04490	5.32
unknown protein	At1g43910	4.20
hypothetical protein predicted by genemark.hmm	At3g13950	4.99
putative cytochrome P450	At3g26830	8.24
hypothetical protein predicted by genscan+	At1g13470	6.96
beta-1,3-glucanase	At3g57240	11.80
serine/threonine kinase	At4g23310	8.78
putative phosphoribosylanthranilate transferase	At4g00700	8.71
putative monodehydroascorbate reductase (NADH)	At3g09940	7.50
putative calcium-binding protein	At3g47480	9.32
disease resistance protein Hcr2-5b	At3g23110	10.30
hypothetical protein	At1g21240	10.03
putative mitochondrial protein	At3g28510	9.84
disease resistance protein	At3g25010	9.96
putative disease resistance protein	At3g11010	8.97
unknown protein	At1g14870	9.04
thaumatin-like protein	At1g75040	8.80
unknown protein	At1g02450	8.25
peptide transporter	At5g46050	6.64
calmodulin-like protein	At3g50770	6.11
protein kinase	At4g11890	7.69
hypothetical protein	At1g35230	7.50
auxin-responsive - Nt-gh3 like	At5g13320	7.11
2,3-oxidosqualene-triterpenoid cyclase	At1g66960	7.86
light repressible receptor protein kinase	At1g51890	8.31
putative vacuolar sorting receptor	At1g30900	8.31
serine threonine kinase	At4g23140	10.99
flavanone 3-hydroxylase-like protein	At5g24530	16.73
endochitinase isolog	At2g43570	14.28
pathogenesis-related PR-1-like protein	At2g14610	15.81
putative receptor-like protein kinase	At4g04500	12.92
putative luminal binding protein	At1g09080	12.85
3(2),5-bisphosphate nucleotidase	At5g64000	11.98
nucleoid DNA-binding protein cnd41	At5g10760	11.52

putative protein CGI-131 protein	At4g21850	12.80
putative disease resistance protein	At2g32680	12.47
embryonic abundant protein EMB34	At3g54150	2.36
similar to MAP kinase 5	At1g01560	2.45
unknown protein	At5g22530	5.83
unknown protein	At5g55420	5.22
metalloendopeptidase NRD2 convertase	At3g57460	5.50
amino acid permease	At4g35180	5.13
similarity to DNA-binding protein zyxin	At3g13610	6.19
SAM:trans-caffeoyl-Coenzyme A 3-O-methyltransferase	At1g67980	5.70
protein kinase	At1g35710	5.95
isochorismate synthase (icsI)	At1g74710	5.37
receptor-like protein kinase RLK3	At3g45860	5.99
putative protein	At3g60470	5.58
unknown protein	At1g72060	5.65
similar to dimethylaniline monooxygenase	At1g19250	5.78
putative retroelement pol polyprotein	At2g04460	5.85
BCS1 - like protein	At5g17760	4.46
unknown protein	At5g55450	4.46
hypothetical protein	At3g28580	4.60
Expressed protein	At1g73800	4.27
putative glutathione S-transferase	At1g74590	4.36
fructosidase	At5g11920	4.35
similar to AtMRP4	At3g13100	4.37
similar to CYTOCHROME P450 76C2	At1g33720	4.38
Ca2+/H+-exchanging protein	At3g51860	4.94
mitochondrial inner membrane translocase	At1g20350	4.93
similar to calmodulin-binding protein	At1g73805	5.09
similar to putative glutamate receptor	At3g07520	5.41
hypothetical protein	At1g10340	5.21
serine/threonine-specific protein kinase	At5g59670	3.80
ornithine cyclodeaminase	At5g52810	3.97
putative chitinase	At4g01700	3.83
putative protein	At5g50200	4.14
frnE protein - like frnE protein	At5g38900	4.06
hypothetical protein	At3g48080	4.31
hypothetical protein predicted by genscan+	At3g18250	4.19
putative phytochelatin synthetase	At5g60950	4.44
putative protein extensin	At4g25110	4.27
putative protein prib5	At3g60420	3.96
unknown protein	At1g08050	3.87
ankyrin-repeat-containing protein-like	At5g54610	4.08
beta-glucosidase	At5g24540	4.14
putative WRKY-type DNA binding protein	At2g25000	4.09
unknown protein	At2g26400	5.00
putative protein	At3g51330	4.79
transport protein SEC61 BETA SUBUNIT	At3g60540	4.72
similar to zeatin O-xylosyltransferase	At3g11340	4.73
putative disease resistance protein	At2g24160	4.70
putative protein	At5g10380	3.88
receptor serine/threonine kinase PR5K	At4g18250	4.33
unknown protein	At5g52740	4.65

similar to unknown protein	At5g64510	4.41
putative L-ascorbate oxidase	At4g39830	4.29
similar to reticuline oxidase-like protein	At1g26380	4.49
putative protein	At5g66640	3.16
hypothetical protein	At4g23610	2.91
nodulin	At5g25260	3.09
S-receptor kinase homolog 2	At5g60900	3.33
serine/threonine kinase	At4g23220	3.29
hypothetical protein predicted by genemark.hmm	At3g28540	3.44
putative glutathione S-transferase	At2g29460	3.40
Phospholipase like protein	At4g38560	3.12
hypothetical protein	At4g03450	2.91
putative protein	At3g52430	3.85
putative DNA damage-inducible protein	At4g39030	3.78
hypothetical protein	At3g50480	3.73
putative alcohol dehydrogenase	At2g47130	3.78
putative ligand-gated ion channel protein	At2g29120	3.67
similar to reticuline oxidase-like protein	At1g26420	3.45
unknown protein	At2g18690	3.54
protein kinase	At1g66880	3.53
nodulin-like protein	At2g39210	3.43
putative glucosyltransferase	At2g30140	3.49
farnesylated protein GMFP5	At5g60800	4.80
N-hydroxycinnamoyl benzoyltransferase-like protein	At5g42830	4.48
hypothetical protein	At1g03850	3.91
dnaK-type molecular chaperone hsc70.1	At5g02490	3.63
unknown protein	At3g26470	3.62
leucine-rich repeat disease resistance protein	At3g24900	3.61
putative DnaJ protein	At3g08970	3.71
catechol O-methyltransferase	At1g33030	3.55
similar to receptor protein kinases	At4g04220	3.41
similar to calreticulin	At1g08450	3.33
unknown protein	At5g46230	3.28
unknown protein Plant lipid transfer protein	At3g22600	3.27
putative aspartate aminotransferase	At2g13810	3.42
putative protein	At5g03350	4.30
alpha-amylase	At4g25000	3.62
feebly-like protein	At3g01420	3.12
UDP-glucose glucosyltransferase	At1g22400	3.21
putative vacuolar sorting receptor	At2g34940	3.05
serine threonine kinase	At4g23130	2.82
hypothetical protein	At1g21250	2.72
endochitinase-like	At3g47540	2.87
hypothetical protein	At4g20000	2.92
cytochrome P450	At3g26320	3.01
receptor serine/threonine protein kinase	At5g38240	2.80
unknown protein	At1g66690	2.88
disease resistance protein EDS1	At3g48090	2.95
unknown protein	At3g26440	2.96
putative heme oxygenase	At1g69720	3.00
WRKY-like protein	At5g13080	2.75
receptor kinase-like protein (Xa21)	At3g47090	2.64

receptor protein kinase	At1g51800	2.59
sucrose transport protein SUC1	At1g71880	2.63
protein phosphatase type 2C	At1g34750	2.64
putative protein	At5g22540	2.42
cytochrome P450 monooxygenase (CYP71B3)	At3g26220	2.41
putative protein	At5g08240	2.38
unknown protein	At1g15790	2.51
putative zinc transporter	At1g05300	2.50
unknown protein	At3g01290	2.46
unknown protein	At2g38860	2.46
Expressed protein	At1g55910	2.72
3'(2'),5'-bisphosphate nucleotidase	At5g09290	2.76
similarity to disease resistance protein	At5g45000	2.80
disulfide isomerase-related protein	At1g04980	2.84
NAC domain protein Tobacco elicitor-responsive gene	At3g44350	2.97
hypothetical protein	At2g04070	3.00
amino acid permease	At5g40780	2.82
protein disulfide-isomerase	At3g54960	2.85
unknown protein	At1g23840	2.81
expansin (At-EXP1)	At1g69530	3.19
serine/threonine kinase	At4g23210	3.09
beta-fructofuranosidase 1	At3g13790	3.14
ABC transporter	At1g30410	2.96
putative protein	At5g53110	3.09
osmotin precursor	At4g11650	3.05
ABC transporter	At1g15520	3.02
putative protein	At4g29520	3.31
putative protein	At5g43910	3.31
putative mutT domain protein	At2g04430	3.32
similar to wild oat DNA-binding protein ABF2	At4g01720	3.17
putative tropinone reductase	At2g29350	3.22
putative ligand-gated ion channel protein	At2g29110	3.19
wall-associated kinase 4	At3g57700	2.45
putative pectinacetylesterase	At3g09410	2.53
unknown protein	At1g55210	2.52
receptor-like protein kinase precurso	At5g58940	2.32
receptor protein kinase	At4g08850	2.32
beta-glucosidase-like protein	At3g60140	2.38
ZIP4, a putative zinc transporter	At1g10970	2.33
thioredoxin	At1g45145	2.32
similarity to seed protein B32E	At1g76970	2.53
pEARLI 4	At4g35110	2.40
EspB-like protein	At5g53550	2.44
hypothetical protein predicted by genemark.hmm	At1g72240	2.44
receptor-like protein kinase	At1g51860	2.28
putative protein embryo-abundant protein (EMB34)	At4g22530	2.30
serine/threonine kinase	At4g23240	2.35
hypothetical protein	At2g12290	2.35
HSP like protein	At4g16660	2.43
calnexin	At5g61790	2.35
putative protein HIRA interacting protein 4 (dnaJ-like)	At3g62600	2.39
unknown protein	At1g15670	2.37

similar to non-phototropic hypocotyl 3	At1g52770	2.36
luminal binding protein	At5g28540	2.26
putative Na+/H+-exchanging protein	At4g23700	2.30
receptor kinase-like protein	At5g47850	2.19
putative protein	At5g37740	2.17
hypothetical protein predicted by genscan+	At3g09490	2.17
glutamine:fructose-6-phosphate amidotransferase	At3g24090	2.19
hevein-like protein precursor (PR-4)	At3g04720	2.20
receptor-like serine/threonine protein kinase ARK3	At4g21380	2.16
similar to harpin-induced protein hin1	At2g35980	2.13
receptor protein kinase	At1g51790	2.32
beta-1,3-glucanase class I precursor	At4g16260	2.22
putative cytochrome P450	At2g45510	2.25
ubiquitin-like protein	At5g55170	2.94
glucosyltransferase IS10a, salicylate-induced, Nicotiana tabacum,	At3g53150	2.89
Expressed protein	At4g00955	2.82
disease resistance protein	At3g25020	2.76
hypothetical protein	At1g21310	2.78
disease resistance RPS2 like protein	At4g14610	2.72
putative RING zinc finger protein	At2g42360	2.67
HSP90-like protein HSP90 homolog	At4g24190	2.50
hypothetical protein	At1g34420	2.51
peroxidase	At5g64120	2.63
unknown protein	At2g02810	2.64
putative cytochrome P450	At2g30770	2.58
NAC-domain protein-like	At5g22380	3.07
hypothetical protein	At3g48640	3.43
ADP,ATP carrier-like protein	At4g28390	3.54
unknown protein	At2g14560	3.44
putative pectinesterase	At2g26440	2.95
putative protein	At5g67340	3.07
cytochrome P450	At3g26210	3.13
putative thioredoxin	At1g77510	3.60
hypothetical protein	At2g40750	3.50
mucin -like protein hemomucin	At3g51440	3.33
hypothetical protein predicted by genemark.hmm	At1g21520	3.48
vacuolar sorting receptor-like protein BP-80	At4g20110	3.24
putative receptor-like protein kinase	At2g19190	3.13
Expressed protein	At4g14365	2.03
heat-shock protein	At5g52640	2.47
serine/threonine kinase	At4g23320	2.32
putative ABC transporter	At1g71330	2.72
hypothetical protein predicted by genemark.hmm	At1g13340	2.68
NAM (no apical meristem)-like protein	At2g43000	2.59
putative protein	At5g24210	2.28
ethylene-forming-enzyme-like dioxygenase-like protein	At5g20400	2.40
putative protein Mlo-h1 protein	At3g45290	2.36
similar to brassinosteroid insensitive 1	At1g74360	2.24
hypothetical protein	At3g22060	2.36
receptor kinase	At1g67520	2.43
glutathione S-transferase	At1g02930	2.06
putative receptor-like protein kinase	At2g31880	2.15

putative reticuline oxidase-like protein	At1g30700	2.02
auxin-induced protein	At4g38840	2.17
putative peroxidase ATP2a	At2g37130	2.04
unknown protein	At1g13750	2.73
disease resistance protein RPP1-WsB	At1g17600	2.63
beta-D-glucan exohydrolase	At5g20950	2.41
putative protein	At4g32870	2.36
unknown protein	At3g29240	2.38
disease resistance protein	At1g17610	2.45
hypothetical protein predicted by genscan	At2g27660	2.48
hypothetical protein similar to pectinesterase	At4g02330	2.46
putative trypsin inhibitor	At1g73260	2.50
SRC2-like shock protein	At3g62780	2.19
putative inositol 1,4,5-trisphosphate 5-phosphatase	At1g05630	2.17
predicted protein predicted protein ERG25	At4g22753	2.11
flower development protein cycloidea (cyc3)	At1g53230	2.10
putative protein MtN21	At5g40230	2.05
putative protein	At5g45510	2.10
putative DNA-binding protein RAV2	At2g36080	2.11
unknown protein	At2g21850	2.21
disease resistance like protein TMV	At5g36930	2.31
putative wall-associated kinase 2	At1g16260	2.32
putative protein prib5	At3g60450	2.25
putative protein	At5g40690	2.21
putative protein annexin VII	At4g23480	2.20
pEARLI 1-like protein	At4g12470	2.29
hypothetical protein predicted by genscan	At2g45760	2.24
amino acid transport protein AAT1	At4g21120	2.17
putative protein P58 protein	At5g03160	2.09
hypothetical protein	At4g14400	2.12
unknown protein	At1g70690	2.13
prolyl endopeptidase	At1g20380	2.07
prolyl 4-hydroxylase	At3g28480	2.05
hypothetical protein	At4g14390	2.13
putative phospholipase D-gamma phospholipase	At4g11840	2.11
serine carboxypeptidase	At3g12220	2.09
hypothetical protein	At2g46150	2.06
unknown protein	At5g24110	2.15
putative receptor protein kinase	At4g23260	2.08
disease resistence - like protein RPP1-WsA / WsC	At3g44400	2.07
cyclic nucleotide and calmodulin-regulated ion channel	At1g01340	2.09
cytochrome P450	At4g39950	2.22
subtilisin-like serine protease	At1g32960	2.19
similarity to endo-beta-N-acetylglucosaminidase	At5g05460	2.08
disease resistance protein	At3g14470	2.12
chitinase	At1g02360	2.16
hypothetical protein	At1g21270	2.01
putative iron-regulated transporter	At1g60960	2.03
similarity to integral membrane protein	At5g54860	2.09
tryptophan synthase alpha chain	At3g54640	2.08
putative indole-3-glycerol phosphate synthase	At2g04400	2.11
similarity to nucellin	At1g49050	2.07

hypothetical protein	At3g21520	2.03
unknown protein	At1g14360	2.03
similar to PEPTIDE TRANSPORTER PTR2-B	At1g68570	2.06
putative glutathione S-transferase	At2g02930	2.11
phosphatase	At4g23570	2.00
unknown protein	At1g68620	2.03
unknown protein	At3g02840	0.48
similar to WRKY transcription factor	At1g80840	0.37
unknown protein	At1g80130	0.18
starch synthase	At1g32900	0.25
Expressed protein	At5g16570	0.21
salt-tolerance zinc finger protein	At1g27730	0.37
unknown protein	At3g25780	0.39
stress related protein	At1g67360	0.47
disease resistance protein	At1g72940	0.45
unknown protein	At1g23710	0.34
putative WRKY-type DNA binding protein	At2g38470	0.41
putative protein AT.I.24	At4g33040	0.46
Nitrilase 4	At5g22300	0.22
late embryogenesis abundant protein	At1g61340	0.13
CCR4-associated factor-like protein	At5g22250	0.26
putative c2h2 zinc finger transcription factor	At5g04340	0.32
putative lipoxygenase	At1g72520	0.21
lipoxygenase	At1g17420	0.23
ethylene-responsive element binding factor	At1g28370	0.17
DRE CRT-binding protein DREB1C	At4g25470	0.28
transcriptional activator CBF1	At4g25490	0.30
Expressed protein	At1g05575	0.30
MtN3-like protein	At5g50800	0.20
anthocyanin 5-aromatic acyltransferase	At5g61160	0.16
Expressed protein	At4g27657	0.14
hypothetical protein predicted by genemark.hmm	At1g60190	0.09
antifungal protein-like (PDF1.2)	At5g44420	0.31
cinnamoyl CoA reductase	At5g14700	0.34
hypothetical protein	At3g04640	0.38
unknown protein	At2g38790	0.34
putative protein	At3g46620	0.44
putative protein retinal glutamic acid-rich protein	At5g28630	0.43
glutaredoxin	At3g62960	0.45
hypothetical protein	At1g20510	0.44
putative L-asparaginase	At3g16150	0.47
transcription factor-like protein	At5g06510	0.43
ethylene responsive element binding factor 4 (AtERF4) identical to GB:BAA32421 from [Arabidopsis thaliana];supported by full-length cDNA: Ceres:22775.	At3g15210	0.43
protein kinase-like protein	At5g67080	0.46
putative C2H2-type zinc finger protein	At2g37430	0.47
glutaredoxin	At5g11930	0.43
putative protein EREBP-3 homolog	At3g50260	0.48
similarity to diphosphoinositol polyphosphate phosphohydrolase	At1g73540	0.41
pyruvate kinase	At3g49160	0.50
putative phi-1-like phosphate-induced protein	At4g08950	0.44
TINY-like protein	At1g21910	0.35

phosphate-induced (phi-1) protein	At1g35140	0.28
hypothetical protein predicted by genemark.hmm	At1g50040	0.41
TCH4 protein	At5g57560	0.29
putative protein	At4g37240	0.43
thionin Thi2.2	At5g36910	0.45
hypothetical protein predicted by genefinder	At1g67910	0.48
unknown protein	At3g19680	0.35
unknown protein	At2g42870	0.45
vegetative storage protein Vsp1	At5g24780	0.24
myb-related protein	At4g37260	0.24
putative protein MEL-26	At3g48360	0.10
putative protein	At4g17470	0.28
putative protein	At5g44260	0.25
putative protein auxin-induced protein 6B	At3g53250	0.19
unknown protein	At2g20670	0.17
putative helix-loop-helix DNA-binding protein	At1g73830	0.31
RING-H2 finger protein RHA3a	At1g49210	0.31
conglutin gamma	At5g19120	0.35
putative tropinone reductase	At2g29300	0.36
putative PR-1 protein	At4g25780	0.32
low-temperature-induced protein 78	At5g52310	0.34
hypothetical protein	At4g10910	0.33
putative protein	At4g04630	0.32
unknown protein	At5g57760	0.35
putative tetracycline transporter protein	At2g16990	0.34
hypothetical protein predicted by genscan+	At1g54740	0.41
cold-regulated protein cor15a precursor	At2g42540	0.37
putative protein	At5g42200	0.39
putative DnaJ protein	At2g17880	0.40
tyrosine transaminase like protein	At4g23600	0.39
unknown protein	At3g15450	0.42
UDP-glucose glucosyltransferase	At1g22370	0.44
Expressed protein	At1g69523	0.43
similarity to homeodomain leucine zipper protein	At5g06710	0.43
ent-kaurene synthase	At1g79460	0.43
unknown protein	At2g15830	0.38
Expressed protein	At5g50335	0.36
unknown protein	At2g44130	0.35
sulfate transporter	At5g10180	0.49
unknown protein	At1g16850	0.42
PGPD14 protein	At5g22920	0.39
unknown protein	At3g07350	0.41
glutaredoxin	At3g62950	0.44
unknown protein	At3g26960	0.43
auxin-induced protein	At1g29500	0.43
putative protein	At5g25240	0.44
putative auxin-induced protein AUX2-11	At1g04240	0.42
At14a-1 protein	At3g28290	0.47
hypothetical protein	At2g36050	0.49
glutaredoxin	At4g15660	0.49
putative protein	At5g64190	0.49
auxin-induced protein	At1g29510	0.48

glucuronosyl transferase-like protein	At5g05890	0.44
Expressed protein	At1g29395	0.46
cytochrome P450	At5g45340	0.15
xyloglucan endo-transglycosylase	At3g44990	0.14
growth factor like protein antisense basic fibroblast growth factor Atnudt7	At4g12720	0.03
ethylene-responsive element binding protein homolog	At4g34410	0.03
hypothetical protein predicted by genscan	At2g34600	0.14
heat shock transcription factor HSF1	At3g24520	0.18
pectinacetylesterase	At1g57590	0.21
anthocyanin2	At1g56650	0.10
glutathione S-transferase-like protein	At5g17220	0.09
hypothetical protein	At1g61890	0.14
putative protein	At5g66650	0.22
Expressed protein	At4g27652	0.18
similar to Anthocyanin 5-aromatic acyltransferase	At1g03495	0.12
Myb-related transcription factor	At1g66390	0.09
expressed protein supported by cDNA: Ceres:13917.	At4g19430	0.18
ethylene responsive element binding factor-like protein (AtERF6)	At4g17490	0.23
putative mitochondrial uncoupling protein	At4g24570	0.28
ethylene responsive element binding factor 5	At5g47230	0.24
unknown protein	At2g42760	0.26
unknown protein	At1g17380	0.18
hypothetical protein predicted by genefinder	At2g22880	0.15
hypothetical protein	At4g01390	0.20
putative protein phosphatase 2C	At2g30020	0.18
dihydroflavonol 4-reductase	At5g42800	0.07
Expressed protein	At4g27654	0.05
flavanone 3-hydroxylase	At3g51240	0.16
flavonol 3-O-glucosyltransferase-like	At5g54060	0.13
AP2 domain containing protein	At1g74930	0.10
allinase	At1g34040	0.07
putative two-component phosphorelay mediator	At3g16360	0.13
putative calmodulin similar to calmodulin	At1g76650	0.12
chalcone synthase	At5g13930	0.12
glucosyltransferase like protein	At4g14090	0.14
anthocyanidin synthase	At4g22870	0.12
disease resistance protein RPS4	At1g65390	0.16
unknown protein	At2g18210	0.18
putative protein dihydrofolate reductase	At4g24380	0.26
putative myo-inositol 1-phosphate synthase	At2g22240	0.23
hypothetical protein	At4g29780	0.17
unknown protein	At1g74450	0.20
non-consensus CG donor splice site at exon 1	At5g56980	0.22
similarity to lectin	At1g65400	0.20
putative thiamin pyrophosphokinase	At5g19470	0.23
putative ethylene responsive element binding factor	At5g51190	0.23
AP2 domain transcription factor	At1g19210	0.23
putative protein	At5g62520	0.31
Anthocyanin 5-aromatic acyltransferase	At3g29590	0.33
flavonoid 3-hydroxylase	At5g07990	0.28
Similar to Nicotiana tumor-related protein	At1g09950	0.26
unknown protein	At5g43150	0.30

thaumatin-like protein	At4g36010	0.30
unknown protein	At3g16860	0.30
beta-VPE	At1g62710	0.28
putative myrosinase-binding protein	At2g39330	0.32
unknown protein	At3g19970	0.36
12-oxophytodienoate-10,11-reductase	At2g06050	0.37
putative protein COP1-interacting protein CIP8	At5g59550	0.39
similar to Cys2/His2-type zinc finger protein 2	At3g19580	0.39
unknown protein	At2g27310	0.41
hypothetical protein	At2g15020	0.41
putative zinc finger transcription factor (PEI1)	At3g55980	0.34
CCR4-associated factor 1-like protein CAF1	At3g44260	0.30
putative protein	At5g59050	0.29
bHLH protein	At1g32640	0.31
putative ethylene response element binding protein	At2g44840	0.31
hypothetical protein predicted by genefinder	At2g43500	0.44
unknown protein	At1g32920	0.46
putative protein	At3g53980	0.48
Expressed protein	At4g04955	0.48
phosphoprotein phosphatase	At3g62260	0.49
putative protein kinase	At2g30040	0.49
scarecrow-like 13 (SCL13)	At4g17230	0.48
ethylene response factor 1 (ERF1)	At3g23240	0.48
scarecrow-like 11 -	At5g59450	0.50
putative nematode-resistance protein	At2g40000	0.49
dehydrodolichyl diphosphate	At5g58770	0.45
sigma-like factor (emb CAA77213.1)	At5g24120	0.45
putative DNA binding protein	At3g57540	0.42
glucose-1-phosphate adenylyltransferase (APL3)	At4g39210	0.46
putative protein	At4g20170	0.45
dof zinc finger protein	At1g51700	0.47
chorismate mutase	At5g22630	0.44
12-oxophytodienoate reductase (OPR2)	At1g76690	0.43
unknown protein	At1g19180	0.45
unknown protein	At2g39650	0.47
transcriptional activator CBF1-like protein	At4g25480	0.29
predicted protein	At1g20823	0.28
Aspartate-glutamate racemase family	At1g15410	0.29
water stress-induced protein	At1g56600	0.34
calcium-dependent protein kinase	At3g57530	0.34
putative trehalose-6-phosphate phosphatase	At2g22190	0.37
putative protein kinase CLV1 receptor kinase	At4g10390	0.35
putative protein	At5g15190	0.32
putative protein	At5g65040	0.34
hypothetical protein	At1g79270	0.34
putative leucine zipper-containing protein	At5g59730	0.29
unknown protein	At5g65300	0.32
GRAB1-like protein	At1g77450	0.33
unknown protein	At1g64360	0.30
AP2 domain containing protein RAP2.4	At4g28140	0.38
calcineurin B-like protein 1	At4g17615	0.35
NAM-like protein	At1g52890	0.36

UDP glucose:flavonoid 3-o-glucosyltransferase	At5g17050	0.37
putative protein	At5g16200	0.36
ferritin 1 precursor	At5g01600	0.37
unknown protein	At2g36220	0.37
calcium-dependent protein kinase	At5g66210	0.39
unknown protein	At2g41640	0.42
Dr4(protease inhibitor)	At1g73330	0.41
unknown protein	At1g18740	0.43
Expressed protein	At2g25735	0.42
physical impedance induced protein, Zea mays	At4g35985	0.45
ABA-responsive protein	At5g13200	0.41
unknown protein	At1g22160	0.40
unknown protein	At1g72450	0.41
putative monogalactosyldiacylglycerol synthase	At2g11810	0.39
NAC2-like protein	At5g24590	0.45
phosphoethanolamine N-methyltransferase	At1g73600	0.43
early light-induced protein	At3g22840	0.47
putative inorganic pyrophosphatase	At2g18230	0.46
cellulose synthase catalytic subunit	At4g23990	0.50
nodulin-like protein MtN21	At4g08300	0.47
nodulin-like protein	At2g39510	0.47
unknown protein	At5g49280	0.41
floral homeotic protein apetala2-like	At5g67180	0.41
putative protein	At5g05140	0.41
RING-H2 finger protein RHA3b	At4g35480	0.43
similar to MAP kinase kinase 5	At1g73500	0.43
hypothetical protein	At1g11210	0.41
AR781, similar to yeast pheromone receptor	At2g26530	0.36
sulfate transporter ATST1	At3g51895	0.37
jacalin	At1g73040	0.38
hypothetical protein	At1g55760	0.36
similar to N-term half of NAC domain protein NAM	At1g69490	0.38
DNA binding protein EREBP-4	At5g61600	0.41
unknown protein	At1g02390	0.43
unknown protein	At1g76600	0.45
unknown protein	At3g23170	0.46
cytochrome P450	At1g19630	0.46
allergen like protein	At4g17030	0.46
putative protein	At3g52180	0.44
hypothetical protein predicted by genscan	At3g01960	0.45
peroxisomal integral membrane protein	At5g27520	0.49
putative protein	At4g34560	0.47
Expressed protein	At4g33905	0.47
hypothetical protein	At1g76070	0.48
dehydrin RAB18-like protein	At5g66400	0.48
hypothetical Na(+)/H(+) antiporter	At3g53720	0.49
putative protein	At5g13220	0.50
similar to hypothetical protein HYP1	At1g11960	0.50
putative sugar transport protein, ERD6	At1g08920	0.49
putative protein	At3g56880	0.46
unknown protein	At1g68600	0.46
myo-inositol-1-phosphate synthase	At4g39800	0.44

AP2 domain containing protein RAP	At1g22190	0.44
similarity to amino acid permease	At1g08230	0.45
unknown protein	At3g18560	0.43
putative senescence-associated protein 12	At2g17840	0.44
trehalose-6-phosphate synthase	At4g17770	0.37
putative beta-1,3-glucanase	At2g27500	0.35
mutT domain protein-like	At5g47240	0.41
SAM:salicylic acid carboxyl methyltransferase-like protein	At5g55250	0.41
similarity to chalcone-flavonone isomerase	At5g05270	0.40
putative protein centrin	At4g27280	0.42
hypothetical protein	At1g29050	0.39
putative protein	At4g12000	0.37
similar to auxin-induced proteins	At3g09870	0.37
hypothetical protein	At2g40130	0.39
CALMODULIN-RELATED PROTEIN 2	At5g37770	0.42
putative protein	At4g24450	0.41
hypothetical protein	At4g01080	0.40
amino acid transporter AAP4	At5g63850	0.43
putative zinc-binding protein	At1g10560	0.42
unknown protein	At1g19770	0.44
hypothetical protein	At2g46640	0.45
hypothetical protein predicted by genscan	At1g24330	0.46
putative cytochrome P450	At2g34490	0.45
putativeflavin-containing monooxygenase FMO3	At5g61290	0.27
carboxyl terminal protease	At3g57680	0.32
ACC synthase (AtACS-6)	At4g11280	0.31
unknown protein	At1g78070	0.29
protein phosphatase 2C	At1g07430	0.21
En/Spm-like transposon protein	At1g49450	0.24
serine/threonine protein kinase	At5g25110	0.29
nucleotide pyrophosphatase	At4g29700	0.27
Expressed protein	At1g53885	0.28
unknown protein	At1g27030	0.28
putative protein	At5g22460	0.28
unknown protein	At1g27200	0.30
9-cis-epoxycarotenoid dioxygenase	At3g14440	0.24
putative protein	At4g27410	0.30
beta-glucosidase	At4g21760	0.31
similarity to remorin	At5g23750	0.30
putative two-component response regulator protein	At2g40670	0.28
myb-related protein, 33.3K	At5g67300	0.39
transcription factor TINY	At1g01250	0.36
integral membrane protein-like	At5g52050	0.33
beta-ketoacyl-ACP reductase	At3g55310	0.34
hypothetical protein	At1g53870	0.34
hypothetical protein	At1g20450	0.38
phosphoinositide-specific phospholipase C	At3g55940	0.42
hyuC-like protein 5-substituted hydantoins	At4g20070	0.43
hypothetical protein predicted by genefinder	At1g70700	0.41
putative CCCH-type zinc finger protein	At2g19810	0.43
similar to flavin-containing monooxygenase	At1g62560	0.43
putative serine carboxypeptidase I	At2g23000	0.43

beta-amylase	At4g15210	0.49
unknown protein	At1g57990	0.47
protein phosphatase 2C (PP2C)	At3g11410	0.47
RGA-like protein	At5g17490	0.47
UDP-glucose:indole-3-acetate beta-D-glucosyltransferase	At1g05560	0.47
protein phosphatase 2C ABI2 (PP2C	At5g57050	0.47
UDP rhamnose--anthocyanidin-3-glucoside rhamnosyltransferase	At4g27570	0.48
unknown protein	At2g17280	0.48
glutathione S-transferase	At2g29450	0.49
MYB96 transcription factor-like protein	At5g62470	0.48
putative sugar transporter	At4g04750	0.46
putative protein beta-(1-3)-glucosyl transferase_	At5g03760	0.45
putative HLH DNA-binding protein	At3g25710	0.46
squalene monooxygenase	At5g24150	0.47
hypothetical protein	At3g07360	0.47
putative protein	At3g49580	0.47
hypothetical protein	At2g33570	0.46
putative peroxidase	At3g42570	0.49
unknown protein	At2g35860	0.49
putative protein	At4g17550	0.47
putative disease resistance protein	At2g34930	0.48
subtilisin-like serine protease	At2g39850	0.33
hypothetical protein	At1g02660	0.31
putative protein	At5g48850	0.35
putative protein	At4g23870	0.35
proline-rich protein	At1g51090	0.30
isochorismate synthase	At1g18870	0.32
aspartic protease	At1g62290	0.38
flavin-containing monooxygenase	At1g62570	0.37
nonspecific lipid-transfer protein precursor	At5g59310	0.41
putative protein	At5g01520	0.41
cinnamyl-alcohol dehydrogenase ELI3-1	At4g37980	0.43
hypothetical protein	At1g20440	0.43
transcription factor-like protein	At5g07690	0.40
predicted protein	At4g35320	0.42
putative DNA-binding protein	At4g01250	0.42
unknown protein	At2g27830	0.43
putative glucanse	At2g32990	0.37
Myb-related transcription factor	At1g18710	0.49
unknown protein	At1g76020	0.49
unknown protein	At2g12400	0.49
leucine-rich repeats containing protein grr1	At5g25350	0.47
putative protein	At3g55760	0.46
Expressed protein	At2g38465	0.48
putative anthocyanin 5-aromatic acyltransferase	At2g39980	0.50
similarity to phytochrome interacting factor 3	At1g51140	0.38
unknown protein	At3g10940	0.41
unknown protein	At3g20300	0.43
R2R3-MYB transcription factor	At3g50060	0.42
anthocyanin 5-aromatic acyltransferase	At3g29670	0.43
hypothetical protein	At2g40530	0.44
unknown protein	At2g32880	0.43

putative pectate lyase	At3g09540	0.40
putative galactinol synthase	At1g09350	0.39
salicylic acid carboxyl methyltransferase	At3g21950	0.40
hypothetical protein	At1g14250	0.41
unknown protein	At5g45310	0.48
putative protein SPOP	At4g37610	0.47
glutathione S-transferase	At3g03190	0.45
putative glutathione transferase	At1g17190	0.45
tropinone reductase-I	At1g07440	0.47
cytidine deaminase 6 (CDA6)	At4g29610	0.49
putative beta-glucosidase	At2g32860	0.49
