# Supplementary material for: Modulation of redox homeostasis under suboptimal conditions by Arabidopsis nudix hydrolase 7
Source: BMC Plant Biol. 2010 Aug 12;10:173. doi: 10.1186/1471-2229-10-173 (PMC3095304; doi:10.1186/1471-2229-10-173)
Supplement: Additional file 3 — Fig. S2: MAPMAN view of receptor-like kinases altered in the Atnudt7-1 mutant. [file 1471-2229-10-173-S3.PDF]

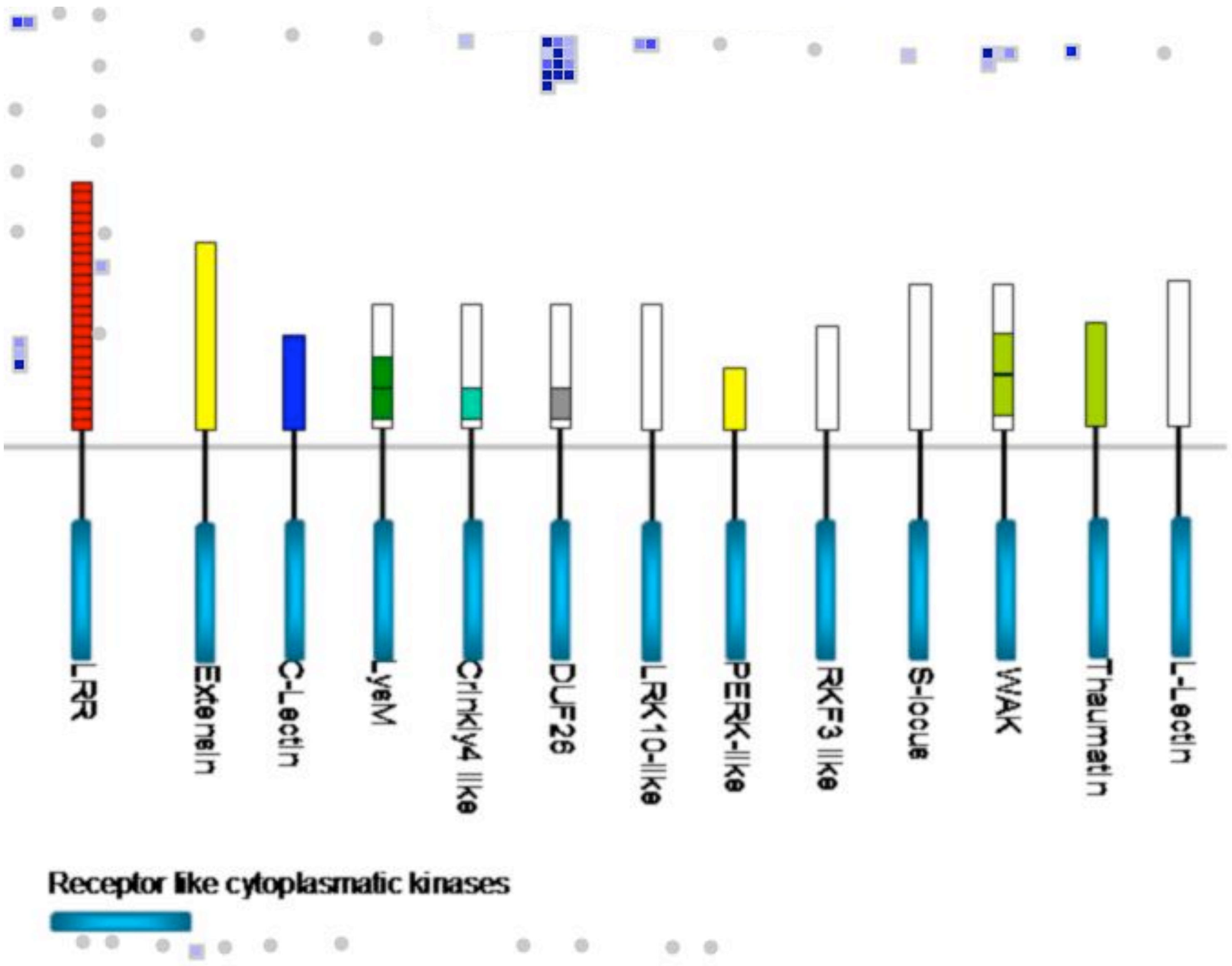

Fig. S2: Induction of large number of Receptor-Like Kinases in the *Atnudt7-1* mutant compared to WT Col-0 plants growing in 12:3:1 mix. This view was generated by the MAPMAN software.
