# Supplementary material for: Modulation of redox homeostasis under suboptimal conditions by Arabidopsis nudix hydrolase 7
Source: BMC Plant Biol. 2010 Aug 12;10:173. doi: 10.1186/1471-2229-10-173 (PMC3095304; doi:10.1186/1471-2229-10-173)
Supplement: Additional file 4 — Table S2: List of primers used for real-time PCR analysis. [file 1471-2229-10-173-S4.PDF]

Table S2. Sequences of the primers used for Real-time PCR analysis

Primer name	Sequence (5’-3’)
At2g46400-F	TCCGTATTTACCAGAATGCCATT
At2g46400-R	TCAAGGCTTCGCTTAAGGATGT
At3g13610-F	AAGCTCTTCCAGAACAATACATTCAG
At3g13610-R	GGAATGGCTTCATCTGTTTCG
At2g41970-F	GGCGGGTAACTTCGGAAAC
At2g41970-R	CCGCAGAAGACACGTCCAT
At1g60190-F	CAAATCCGAGTCGGGTCAAT
At1g60190-R	AGATGACGTGAAGCTCTGAAAGG
At4g26950-F	TGTGTGGGATGTTCTTGATGGA
At4g26950-R	GGGTTGAGAAAGAAGGTTTAGTCGTA
At4g34410-F	GCGCCGTGTCAGGGTTT
At4g34410-R	AATCCAACCGAGGCATTCC
At4g15560-F	GGTACTGCCTGATCGATACATTGA
At4g15560-R	CGATTAGTTAAGTGCGGTTGCT
At2g26500-F	GGTAATGGAGGAGCCTTATCCA
At2g26500-R	GCGTTCATGATTGCTGCAAT
AT2G41530-F	CACCTGCACGGACGAGAAC
AT2G41530-R	GCAATGCCGTGAGTAGAAGCA
Nudt2-F	GATGGCTCAGTCAGAGAGGTG
Nudt2-R	CGCTTTGTGTGTTTGTCTGAA
Nudt10-F	CAGGGTAAAAAGGGAGTCTGG
Nudt10-R	ATGGTGATACCGGAAACCTTC
Nudt6-F	GATTTTGTCCCAAAGCTGAGA
Nudt6-R	CATCGGCAAGCTTTAACCATA
Nudt7-F	ACCATGGAACAAGAAGAACGA
Nudt7-R	GCGAATCCCAAGTATTCTTCC
